# Adaptive management for alpine grassland of the Tibetan Plateau based on a multi-criteria assessment

**DOI:** 10.3389/fpls.2025.1518721

**Published:** 2025-03-12

**Authors:** Tianyu Zhan, Shurong Zhang, Wenwu Zhao

**Affiliations:** ^1^ State Key Laboratory of Earth Surface Processes and Resource Ecology, Faculty of Geographical Science, Beijing Normal University, Beijing, China; ^2^ Institute of Land Surface System and Sustainable Development, Faculty of Geographical Science, Beijing Normal University, Beijing, China

**Keywords:** adaptive management, zone, multi-criteria, alpine grassland, Tibetan Plateau

## Abstract

With the increasing threats of global climate change and human activities to terrestrial ecosystems, understanding the quality of alpine grassland ecosystems and their influencing factors is fundamental for effective ecosystem management and improving human well-being. However, current adaptive management plans for alpine grasslands based on multi-criteria assessment are limited. This study utilized field investigations at 77 sampling points, drone remote sensing, and satellite remote sensing data to construct an alpine grassland quality index based on vegetation and soil indicators, and assess the ecosystem’s resilience and pressure. The assessment revealed that the alpine grasslands of the Tibetan Plateau were classified into five zones, indicating significant differences in quality and pressure levels. Key findings showed that the High-Quality Pressure Zone comprise 41.88% of the area of alpine meadow and 31.89% of alpine steppe, while the Quality Improvement-Limitation Zone account for 21.14% and 35.8% of the respective areas. The study recommends graded protection and recovery strategies for alpine grasslands based on quality levels: prioritizing high-quality grasslands, implementing dynamic monitoring and enhancement for moderate-quality grasslands, and applying artificial interventions and suitable species for low-quality grasslands. This research underscores the importance of zoning-based adaptive strategies for sustainable ecosystem management and provides valuable insights for effective management and protection of alpine grasslands in the Tibetan Plateau.

## 1 Introduction

Climate change (Gao et al., 2014) and grazing activities (Lu et al., 2017) are significant driving forces influencing alpine grassland ecosystems, especially in high-altitude regions where sensitivity and vulnerability are heightened (Li et al., 2020b; Wang et al., 2024a). These impacts have led to substantial declines in grassland quality, exacerbating biodiversity loss (Li et al., 2018b), soil erosion (Li et al., 2019), and reduced carbon sequestration capacity (Wang et al., 2023), representing a pressing global environmental challenge (Dong et al., 2020). In addition to their ecological functions, alpine grasslands play important societal roles, such as supporting grazing and providing livelihoods for local communities (Wang et al., 2024b). Therefore, systematic ecosystem restoration and conservation measures are crucial to enhancing the quality of alpine grasslands (Harzé et al., 2018). This is not merely a local ecological concern but a vital component of global ecological health.

Traditional management paradigms for alpine grasslands primarily focus on static objectives, often guided by a single indicator, such as forage yield (Loucougaray et al., 2015) or vegetation biomass (Jäger et al., 2020), and lack comprehensive consideration of the dynamic changes within the ecosystem (Dong et al., 2022). While this approach may effectively enhance short-term productivity, it frequently overlooks the complexity and diversity of the ecosystem (Chapin et al., 2010), leading to resource overexploitation and ecological imbalance (Zhou et al., 2023). In light of the complexities associated with changes in alpine grassland quality, a singular management strategy is clearly inadequate. Researchers have proposed the adoption of a multi-criteria comprehensive assessment approach to better understand and address the dynamic changes in alpine grassland ecosystems (Villoslada et al., 2018; Grilli et al., 2017). Therefore, the restoration and conservation of alpine grassland quality require two key decision-making foundations: (1) the current state of alpine grassland quality; and (2) the external pressures faced by alpine grasslands (such as climate change and grazing activities) and its resilience.

In response to the increasing complexity and challenges faced by alpine grasslands, resilience theory has provided a new perspective for managing these fragile ecosystems (Yang et al., 2022; Ji et al., 2024). Adaptive management, as a key application of resilience theory, provides a dynamic and responsive framework for conservation and restoration efforts (Stoffels et al., 2024; Garmestani et al., 2023). This approach emphasizes the unique characteristics and evolving conditions of alpine grasslands, allowing for continuous adjustments to management strategies (Wang et al., 2022). Addressing the ongoing pressures from climate change and intensive grazing requires a more comprehensive and environment-specific management framework (Shang et al., 2014). While various restoration techniques, such as fertilization (Jiang et al., 2013), reseeding (Tian et al., 2023), and grazing exclusion (Sun et al., 2020), have been applied in the alpine grassland ecosystems of the Tibetan Plateau, these methods often face challenges. Natural recovery processes remain sluggish (Zhen et al., 2018), and artificial restoration approaches can be technically demanding and resource-intensive (Dong et al., 2015). Furthermore, the short-term benefits of these interventions are limited, with uncertainties surrounding their long-term efficacy and sustainability (Sun et al., 2024; Zhu et al., 2023). Therefore, it is imperative to develop a context-specific management framework that integrates restoration techniques into a coherent strategy, tailored to the unique environmental conditions of alpine grasslands. The significant spatial heterogeneity of alpine grassland ecosystems across different regions further limits the effectiveness of generic management practices, which often fail to adapt to diverse environmental conditions (Wang et al., 2020). A more integrated management framework is essential—one that addresses the distinct types of alpine grasslands while incorporating their current ecological status, external pressures, and intrinsic resilience.

To address these challenges, adaptive zoning management has emerged as a promising regional planning method (Wang et al., 2022). This approach involves implementing tailored regulatory measures for different spatial areas, acknowledging the unique ecological functions and characteristics of each zone (Jiang et al., 2024). For instance, China’s ecological protection red lines classify regions according to the significance of their ecological functions into ecological red line zones and both important and generally important ecological functional areas (Bai et al., 2018). Different zones have varying ecological protection policies. Recent studies on grassland adaptive zoning demonstrate that it is essential to fully consider the impacts of climate change and human activities, highlighting the necessity of effective management strategies (Wang et al., 2022). By recognizing and responding to the inherent variability among alpine grasslands, zoning adaptive management can facilitate more effective conservation strategies that cater to local ecological dynamics.

The TP serves as a vital ecological security barrier in China, playing a critical role in maintaining regional and global environmental stability (Liu et al., 2021). Understanding the current quality, resilience, and external pressures of alpine grassland ecosystems is essential for implementing adaptive management practices tailored to different zones, thereby ensuring the sustainable development of these environments (Huber et al., 2013). This study aims to address the aforementioned challenges by (1) integrating quantitative assessments of alpine grassland quality, resilience, and external pressures to support planning and decision-making, and (2) providing zoning-based conservation and restoration strategies specific to alpine grasslands.

## 2 Materials and methods

### 2.1 Study area

Our sampling sites covered the TP, primarily including regions in Tibet, Qinghai Province, and northwestern Sichuan Province (**Supplementary Figure S1**). Within an altitude range of 3000 to 5000 meters, a grassland transect approximately 5000 kilometers long was sampled for vegetation and soil across the main grasslands of the TP. The transect sample plots were natural zonal grasslands, including alpine meadow (AM) and alpine steppe (AS). A total of 77 sample plots were collected from the alpine grasslands, including 49 AM plots and 28 AS plots (**Supplementary Figure S1**).

### 2.2 Date collection

#### 2.2.1 Field data

Field surveys were conducted during the peak vegetation growth periods of 2021 and 2022 (i.e., between July and August) in various alpine grasslands on the TP, with priority given to areas with uniform vegetation distribution. Sample plots were chosen based on a stratified random sampling method to ensure representativeness across the different vegetation types within the study area. In each selected sample plot, three large quadrats of 30×30 meters were established to record detailed plot attributes. To assess the structure and functional characteristics of the vegetation community, 0.5×0.5m subplots were randomly located within each quadrat. At each site, aboveground biomass was obtained by clipping all vegetation within the selected subplots at ground level using scissors. Soil samples were collected using a soil auger with a diameter of 5 cm at depths of 0-10 cm, 10-20 cm, and 20-30 cm. To minimize the risk of mold in plant samples, species identification and preliminary drying of all samples were completed within 48 hours after collection for subsequent laboratory analysis. Roots were washed free of soil residues with clean water, then dried in an oven at 65°C for about 48 hours until a constant weight was achieved to determine belowground biomass. Plant diversity indices, including the Shannon-Wiener index, Simpson’s index, and Pielou’s index, were calculated using standard methods. Soil bulk density and moisture content were measured using the ring knife method and drying method, respectively. Specifically, soil samples obtained by the ring knife method were first weighed fresh, then dried in an oven at 105°C for 24 hours until a constant weight was reached. Soil organic carbon content was determined using the potassium dichromate oxidation-external heating method, total nitrogen content was measured by the sulfuric acid-hydrogen peroxide digestion-semi-micro Kjeldahl method (Standard NY/T 2419-2013), and total phosphorus content was determined by the sulfuric acid-hydrogen peroxide digestion-molybdenum antimony anti-colorimetry method (Standard DB37/T 1625-2010). Soil available nitrogen was measured by the alkali-hydrolyzed diffusion method, available phosphorus by the molybdenum antimony anti-colorimetry method, and soil pH was determined using a portable soil tester (TDR 100, Spectrum Technologies Inc., Chicago, USA).

#### 2.2.2 UAV remote sensing

The multispectral remote sensing images used in this study were acquired by the DJI Phantom 4 Multispectral drone (DJI-P4M, DJI Technology Co., Ltd., Shenzhen, China). The DJI-P4M is equipped with an integrated multispectral imaging system, which includes one visible light sensor and five multispectral sensors. These sensors cover three visible light bands, one red-edge band, and one near-infrared band. The central wavelengths of the imaging bands are as follows: blue band (450nm ± 16nm), green band (560nm ± 16nm), red band (650nm ± 16nm), red-edge band (730nm ± 16nm), and near-infrared band (840nm ± 26nm). Each sensor has a resolution of 2 megapixels and uses the same global shutter. The entire system is mounted on a 3-axis gimbal. Before data collection, gray card photographs were taken for radiometric calibration, with data collection scheduled between 12:00 and 16:00 to ensure optimal lighting conditions. For calibration and validation of the UAV data, three 50 cm × 50 cm PVC plates with black and white crisscross lines were utilized as positioning plates, evenly laid on the ground in each landscape. The center point of each PVC plate was designated as the image control point, allowing for the accurate recording of latitude and longitude information. To enhance data validity, flight path planning was completed using DJI GS PRO software. The flight area was set to 200m × 200m, with forward and side overlap rates of 90% and 70%, respectively. Data collection was conducted at a flight altitude of 100m with a nadir view angle of 90°.

The raw UAV aerial survey image data were processed indoors to produce usable product data. Initially, the raw images were manually inspected to remove invalid images taken during takeoff and landing. The valid flight data were then processed using Agisoft Photoscan software for stitching and calibration to generate orthomosaic images. The actual sample plot size was 50cm × 50cm, and the spatial resolution of the UAV multispectral images was 3.1cm. Three small sample plots were used to represent a larger plot, and the raster data corresponding to the larger plot were statistically analyzed. Vegetation indices were extracted from the data points by clipping the corresponding large plots. Vegetation indices (VIs) are common and effective indicators in remote sensing ecological studies (Glenn et al., 2008; Xue and Su, 2017). This study utilized five multispectral bands, namely blue, green, red, red-edge, and near-infrared (NIR). Numerous vegetation indices based on these multispectral bands have been constructed and widely used. These indices have proven effective in monitoring plant growth and ecosystem characteristics. In this study, 42 vegetation indices, incorporating the B, G, R, RE, and NIR bands, were selected for the inversion of alpine grassland quality at the landscape scale (**Supplementary Table S2**).

#### 2.2.3 Remote sensing data

The products selected for the inversion of regional alpine grassland quality include NDVI (Normalized Difference Vegetation Index), EVI (Enhanced Vegetation Index), and LAI (Leaf Area Index). The NDVI data, representing vegetation greenness characteristics, were derived from the MODIS Terra MOD13A1 16-day composite data with a resolution of 500 m. The LAI data, representing vegetation cover characteristics, were obtained from the MODIS Terra MOD15A2H product, which provides 8-day composite data with a 500 m spatial resolution. The EVI, indicative of vegetation productivity, was derived from the MODIS Terra MOD13A1 product, which offers 16-day composite data with a 500 m spatial resolution, covering the time frame of August 2021, corresponding to the sampling and UAV aerial photography period.

Vegetation type data were sourced from the 1:1,000,000 scale China Vegetation Type Map provided by the Resource and Environment Science and Data Center (RESDC) of the Chinese Academy of Sciences (http://www.resdc.cn). Using the vector boundary of the TP, vegetation type data were clipped from the region. By aggregating and extracting similar vegetation types, the spatial distribution ranges of AM and AS on the TP were classified. Air temperature and precipitation data for the growing season (May-September) from January 2001 to December 2020 were selected for this study. These data were sourced from the ERA5-Land dataset. The ERA5-Land data have a temporal resolution of 1 hour and a spatial resolution of 0.1° (Dee et al., 2011). The grazing data were collected from the National Tibetan Plateau Data Center (Liu, 2023). The spatial resolution was 1 km and the temporal resolution was year.

### 2.3 Data analysis

#### 2.3.1 Grassland quality assessment

In this study, data from field transects were utilized to select 14 vegetation and soil indicators. Principal Component Analysis (PCA) was employed to filter these indicators, subsequently constructing the plot-scale alpine Grassland Quality Index (GQI). The GQI was developed at three scales: plot, landscape, and regional. By using UAV-based multispectral vegetation indices as an intermediary, field survey data of grassland ecosystem communities were integrated with remote sensing ecological parameters. Through statistical analysis and model computation, relationships between parameters across multiple scales were established, thereby enabling the assessment of the GQI at the regional scale.

##### 2.3.1.1 Plot-scale grassland quality index

To quantitatively assess the ecosystem quality of AM and AS, this study adopted a dual methodological framework that combines data on grassland vegetation and soil attributes. First, key indicators that reflect grassland quality were selected through (PCA), including biomass, vegetation cover, diversity, soil organic carbon, total nitrogen, total phosphorus, and soil moisture. These indicators were chosen because they are essential for understanding the health, productivity, and resilience of grassland ecosystems. By integrating these critical factors, we can better assess the overall grassland quality. After selecting the key indicators, a comprehensive GQI was constructed. The GQI integrates multiple ecological indicators into a single index, simplifying the assessment process and providing an intuitive measure of grassland quality. The construction of the GQI involves standardizing each key indicator and assigning corresponding coefficients based on their weights in the PCA to ensure that the contribution of each indicator to the final index is proportionate to its importance in representing grassland quality (**Supplementary Table S1**).

##### 2.3.1.2 Landscape-scale grassland quality index

To define GQI at the landscape scale, this study employed 42 vegetation indices derived from UAV multispectral imagery as independent variables (**Supplementary Table S2**), with the plot-scale GQI serving as the dependent variable. A stepwise regression analysis was performed to establish the relationship between the plot-scale GQI and the UAV-derived vegetation indices (**Supplementary Table S3**). The significance of each variable was assessed using a Random Forest model (**Supplementary Figure S2**), leading to the formulation of a robust regression relationship for the GQI based on multispectral vegetation indices (**Supplementary Table S4**).

Multivariate Stepwise Regression (MSR) was utilized to identify the most significant predictors among the selected vegetation indices, involving the iterative addition and removal of variables based on their statistical significance, thus optimizing the model to retain only those variables that substantially explain the variability in grassland quality (**Supplementary Table S4**). The criteria for variable inclusion and exclusion were based on the Akaike Information Criterion (Ghani and Ahmad, 2010). Building on the insights gained from the stepwise regression, we implemented a Random Forest (RF) model to refine the selection of key vegetation indices (**Supplementary Figure S1**). The RF algorithm aggregates predictions from multiple decision trees constructed during training, thereby enhancing predictive accuracy and mitigating overfitting. This approach excels in managing the complex interactions between predictor variables (Rigatti, 2017).

To validate the landscape-scale GQI model, seventy percent of the sampling data was designated as the training set, while thirty percent was utilized as the testing set. The model’s accuracy was assessed using the coefficient of determination (R²). The observed values corresponded to the plot-scale GQI, and the predicted values represented the landscape-scale GQI (**Supplementary Figure S3**).

##### 2.3.1.3 Regional-scale grassland quality index

At the regional scale, the landscape-scale GQI served as the ground truth for further inversion of the regional-scale GQI. Using the GQI as the dependent variable, and NDVI, EVI, and LAI as independent variables, a Partial Least Squares Regression (PLSR) model was constructed (**Supplementary Table S5**). PLSR combines the advantages of PCA, Canonical Correlation Analysis (CCA), and Multiple Linear Regression (MLR). While PLSR and PCA aim to extract maximum information reflecting data variation, PCA focuses solely on one matrix of independent variables. In contrast, PLSR simultaneously considers the correlations between independent and dependent variables, thereby enhancing predictive capabilities (Geladi and Kowalski, 1986). Overall, the PLSR algorithm effectively mitigates collinearity among variables and optimizes the use of spectral information, resulting in improved modeling accuracy and estimation outcomes (Geladi and Kowalski, 1986). To evaluate the performance of the regional-scale model, leave-one-out cross-validation (LOOCV) was implemented (**Supplementary Figure S4**) (Kearns and Ron, 1997). The GQI of AM and AS on the TP is categorized into high-quality (HQ), moderate-quality (MQ), and low-quality (LQ) areas based on natural breakpoints.

#### 2.3.2 Resilience assessment

Grassland resilience refers to the ability of grassland ecosystems to recover to their original state after disturbance (Gunderson, 2000). In this study, grassland resilience primarily considers the response of vegetation growth to the rate of climate change. T The methods and data used to assess ecosystem resilience are derived from traditional approaches (Yao et al., 2021), with optimizations made to the linear regression model (Li et al., 2018a). The model can be represented as follows:


(1) $$LAI_{t}=\text{a}*tem_{t}+\text{b}*pre_{t}+c*LAI_{t-1}+k$$

Where *LAI_t_*, *tem_t_*, *pre_t_* represent the *LAI*, temperature, and precipitation sequences at time *t*, respectively, and *LAI_t-1_* denotes the *LAI* value at time *t-1*. The coefficients *a*, *b*, and *c* correspond to temperature, precipitation, and *LAI* from the previous month, with *k* as the regression error. The coefficients a, b, and c were derived through a multiple linear regression analysis, using historical data of LAI, temperature, and precipitation. To ensure comparability and eliminate bias due to differing units, these coefficients are normalized using min-max standardization, transforming their values to a range between 0 and 1.Additionally, using the coefficients *a* and *b* from Equation 1, Equation 2 calculates the sensitivity of the ecosystem to climate variability (Seddon et al., 2016):


(2) SI=a*sens(tem)+b*sens(pre)

In this context, *SI* represents the sensitivity index, while *sens(tem)* and *sens(pre)* denote the ecosystem’s sensitivity to temperature and precipitation changes, quantified through the residuals obtained from linear fits of vegetation variation against temperature and precipitation changes (Seddon et al., 2016). According to Equation 3, we utilize coefficient c to evaluate the recovery capacity of the ecosystem following climate disturbances, indicating that a smaller c value corresponds to stronger recovery capability:


(3) RI=1−c

*RI* refers to the resilience index of the alpine grassland ecosystem. The resilience of AM and AS on the TP is categorized into high-resilience (HR), moderate-resilience (MR), and low-resilience (LR) areas based on natural breakpoints.

#### 2.3.3 Pressure assessment

Pressure factors, such as grazing, precipitation, and temperature, play a critical role in the protection and restoration of alpine grasslands. In this study, pressure factors were determined by standardizing the grazing, precipitation, and temperature data and then aggregating them. To effectively assess the pressure gradients in different regions, the natural breaks method was employed to categorize the pressure factors of the AM and AS on the TP into high-pressure (HP), moderate-pressure (MP), and low-pressure (LP)areas.

### 2.4 Zoning basis

Based on the ecological characteristics of the alpine grassland ecosystems on the TP, this study established adaptive zoning management for alpine meadows and alpine steppes. The zoning criteria were based on the natural break method, utilizing key indicators and methodologies derived from vegetation and soil attributes at the plot scale, alongside spatial assessments of grassland quality at the regional scale. The analysis primarily focused on three critical dimensions: grassland quality, resilience, and pressure factors. Using the layers derived from these three individual indicators, the study employed analytical methods such as threshold segmentation and overlay calculations to identify five distinct zone types: Low-Quality Zone (LQZ), Quality Improvement-Limited Zone (QILZ), Quality Restoration Potential Zone (QRPZ), High-Quality Stable Zone (HQSZ), and High-Quality Pressure Zone (HQPZ) (**Table 1**).

**Table 1:** Zoning based on grassland quality, resilience, and pressure.

Zone Names	Zoning Basis
Low-Quality Zone (LQZ)	Low quality grassland
Quality Improvement-Limited Zone (QILZ)	Low to moderate resilience grasslands in moderate quality
Quality Restoration Potential Zone (QRPZ)	High resilience grasslands in moderate quality
High-Quality Stable Zone (HQSZ)	Low pressure grasslands in high quality
High-Quality Pressure Zone (HQPZ)	Moderate to high pressure grasslands in high quality

## 3 Results

### 3.1 Classification of alpine grassland quality, resilience and pressure

**Figure 1:**
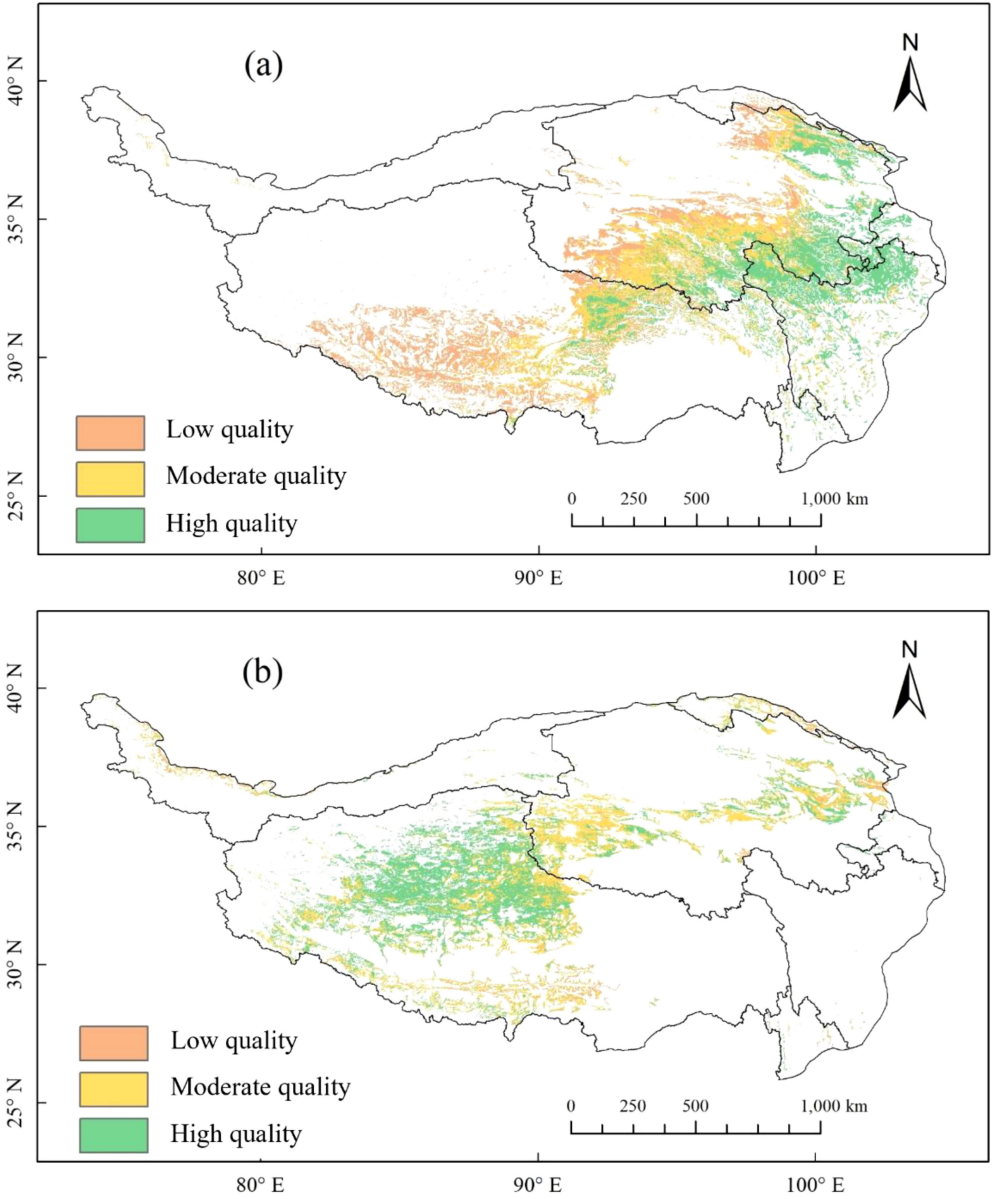
Spatial pattern of alpine grassland quality in Tibetan Plateau from 2001-2020: **(a)** alpine meadows; **(b)** alpine steppes.

**Figure 2:**
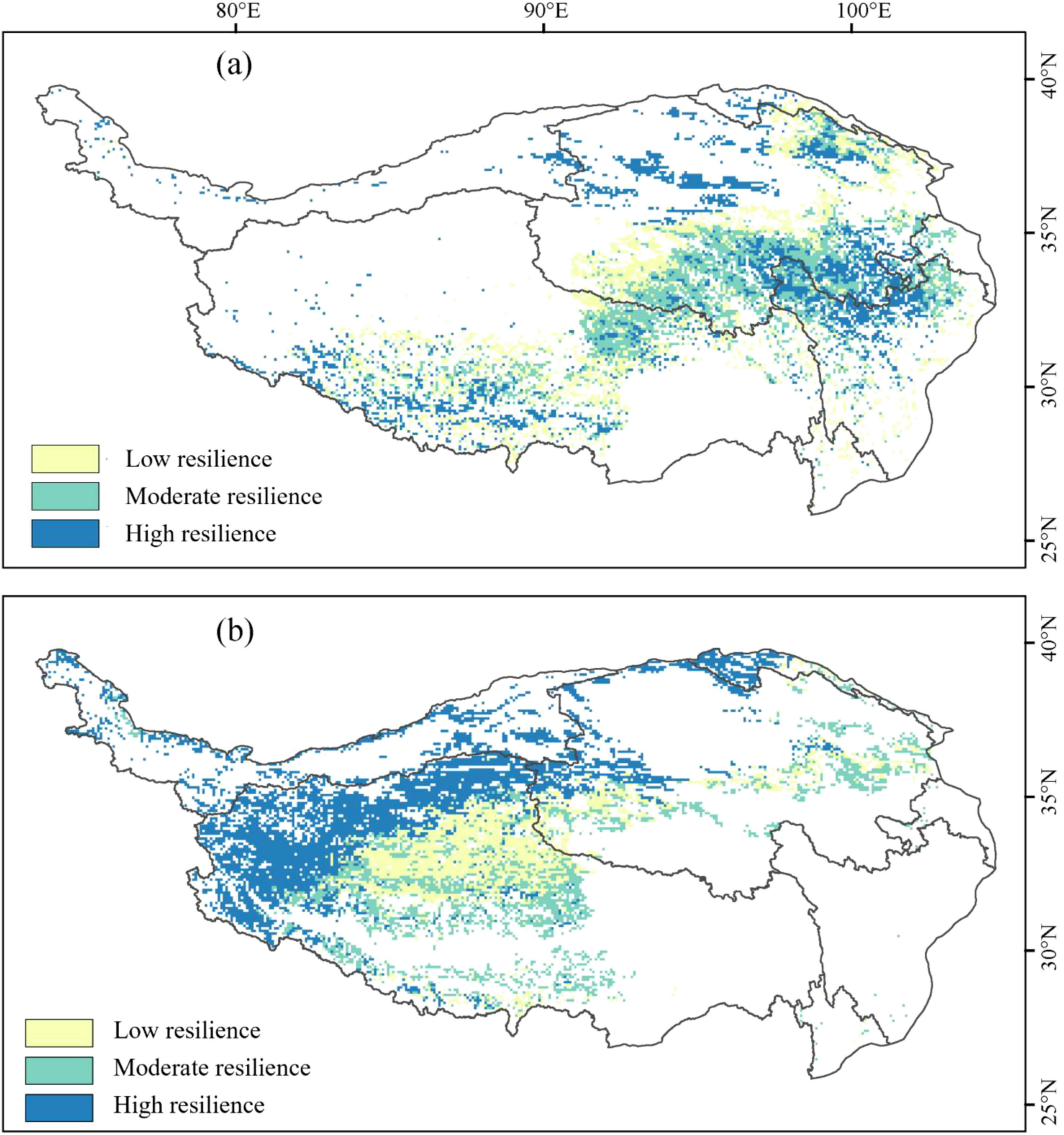
Spatial pattern of alpine grassland resilience in Tibetan Plateau from 2001-2020: **(a)** alpine meadows; **(b)** alpine steppes.

**Figure 3:**
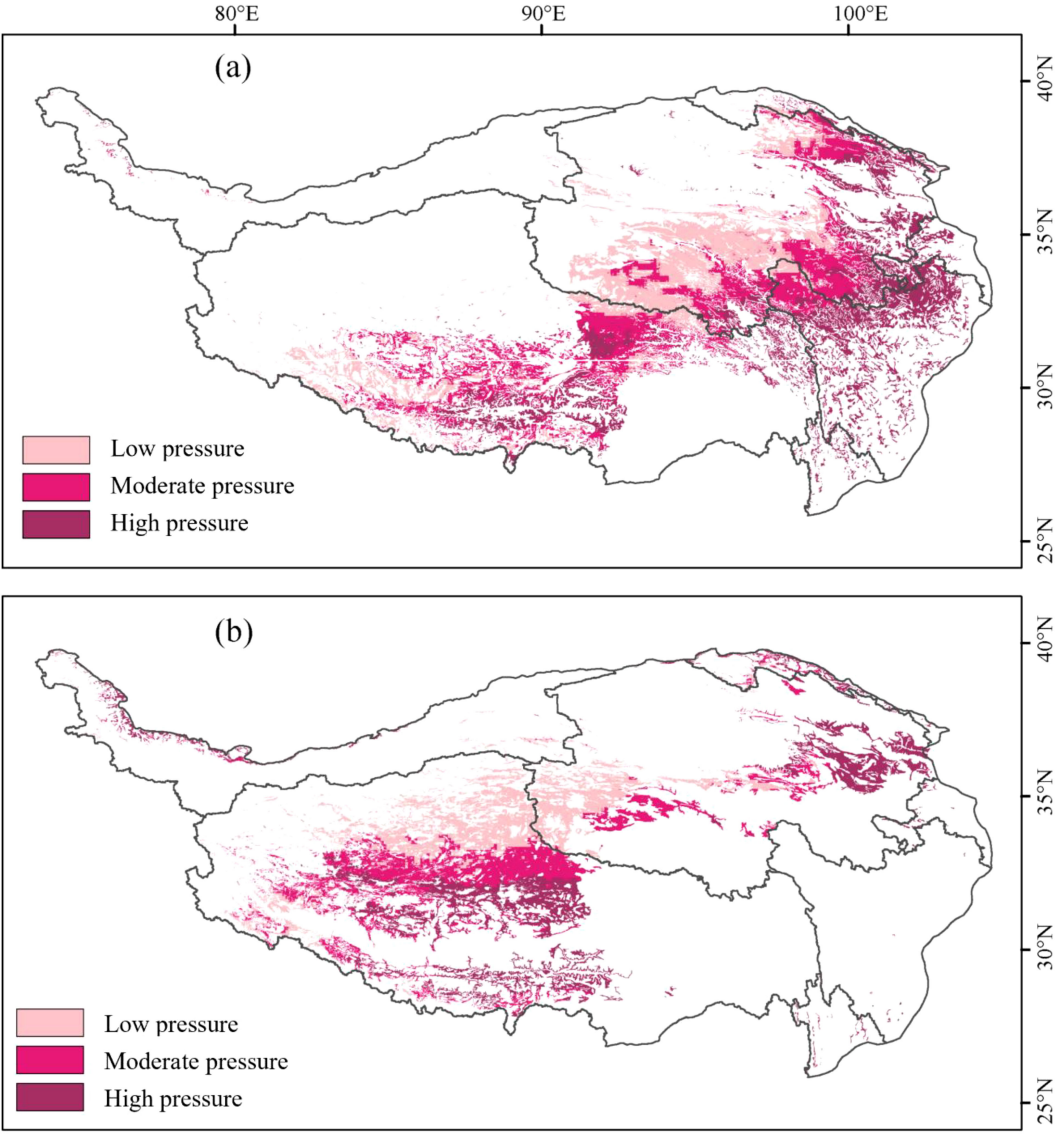
Spatial pattern of alpine grassland pressure in Tibetan Plateau from 2001-2020: **(a)** alpine meadows; **(b)** alpine steppes.

The distribution of grassland quality in the AM of the TP from 2001 to 2020 exhibits significant spatial heterogeneity. LQ meadows, MQ meadows, and HQ meadows account for 33.58%, 34.48%, and 31.93%, respectively. The spatial distribution characteristics show a decreasing trend in the quality of AM from southeast to northwest. In contrast, the spatial distribution characteristics of grassland quality in the AS are markedly different from those in the AM. LQ steppes, MQ steppes, and HQ steppes comprise 3.72%, 40.71%, and 55.57%, respectively. Notably, the quality of AS demonstrates the highest values in the Qiangtang Nature Reserve, with lower quality observed in densely populated areas near Xining and Lhasa (**Figure 1**). Areas of LR, MR and HR in the AM make up 34.34%, 30.33%, and 35.33%, respectively. Areas of LR, MR and HR in the AS account for 24.48%, 25.70%, and 49.82%, respectively. The resilience overall shows a decreasing trend from north to south: the northern region exhibits the strongest resilience, particularly in the central area of the Qiangtang Nature Reserve (**Figure 2**). Areas of LP, MP and HP in the AM consist of 31.63%, 34.07%, and 34.29%, respectively. In the AS, areas of LP, MP and HP make up 32.66%, 33.72%, and 33.62%, respectively. HP areas are primarily concentrated in regions with intense human activities, such as Lhasa, Xining, and northern Sichuan, whereas LP areas are mainly distributed within nature reserves (**Figure 3**).

### 3.2 Adaptive zoning management of alpine grassland

**Figure 4:**
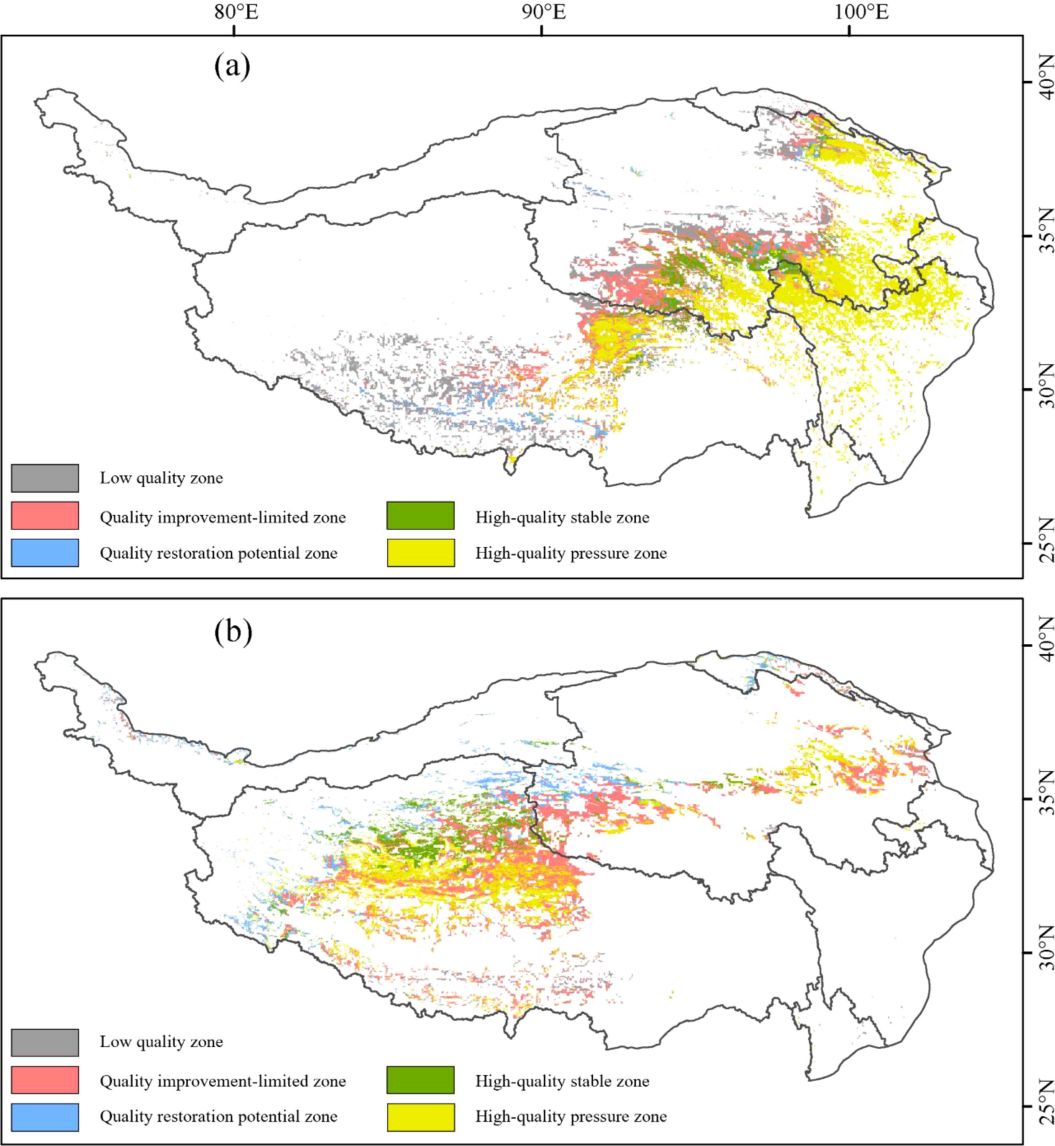
Spatial pattern of alpine grassland zoning in Tibetan Plateau: **(a)** alpine meadows; **(b)** alpine steppes.

Based on the dimensions of grassland quality, resilience, and pressure, five distinct zonations were identified for the AM and AS on the TP. For the AM, LQZ account for 24.26%, QILZ account for 21.14%, QRPZ comprise 4.83%, HQSZ make up 7.89%, and HQPZ represent 41.88%. In the case of the AS, LQZ constitute 4.74%, QIlZ make up 35.80%, QRPZ account for 13.39%, HQSZ comprise 14.18%, and HQPZ cover 31.89% (**Figure 4**). In terms of spatial distribution, QILZ in the AM are mainly found in the Sanjiangyuan region. HQSZ and HQPZ are concentrated in the Qilian Mountain alpine basin. In the AS, HQSZ and HQPZ are primarily located in the Qiangtang Nature Reserve, while the QILZ and QRPZ are spread across the Ali Mountain desert, Qiangtang Nature Reserve, and the Kunlun Mountains.

## 4 Discussion

**Figure 5:**
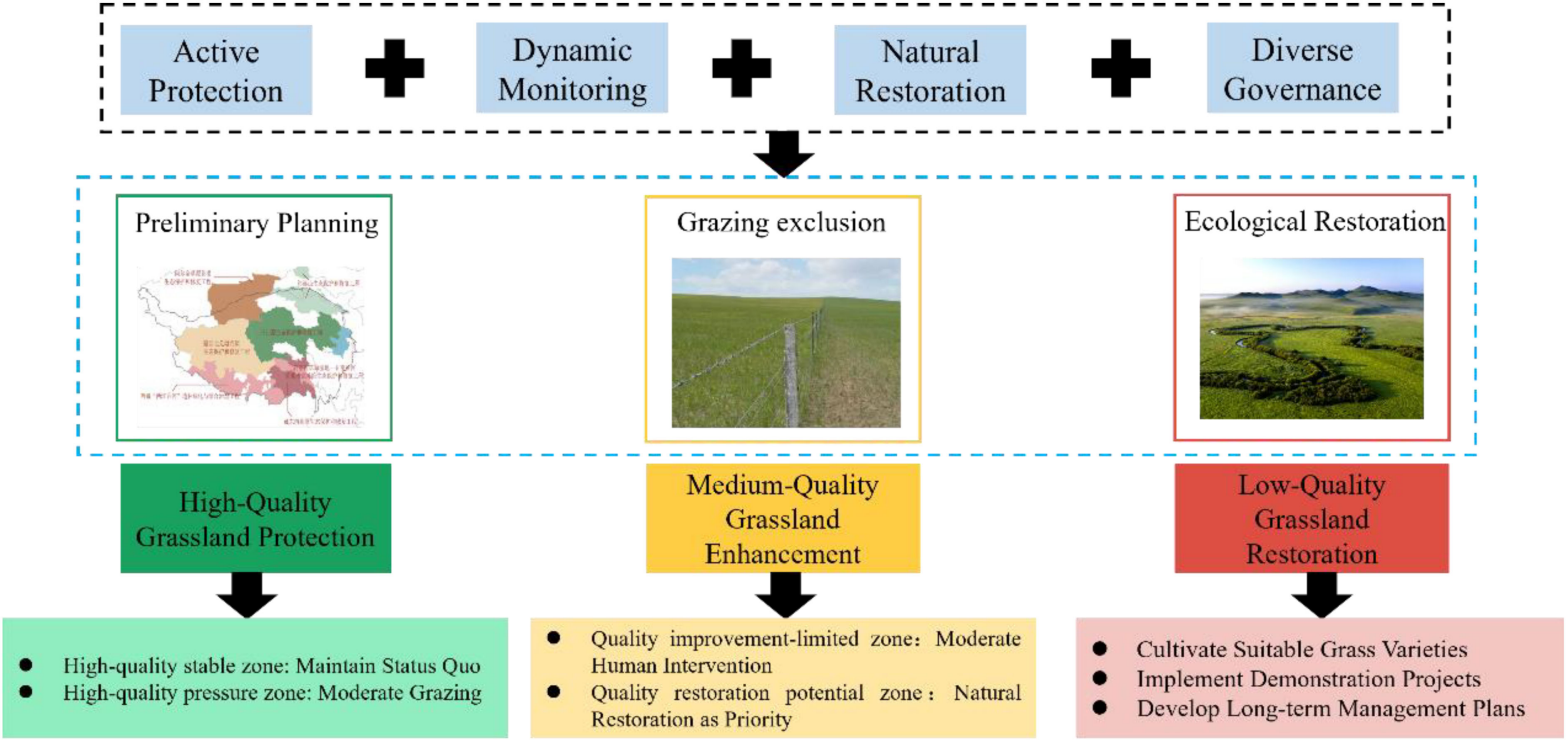
Implementation pathways for the protection and restoration of alpine grassland.

The zoning results of this study are consistent with existing research on the spatial heterogeneity of alpine grasslands on the Tibetan Plateau. For instance, previous studies have also emphasized the significant differences in ecological characteristics across different regions (Sun et al., 2023; Li et al., 2020a). This consistency highlights the robustness and applicability of our framework in a broader ecological context. Additionally, the zoning results provide a comprehensive basis for grassland protection and restoration, facilitating a deeper understanding of the patterns and processes governing alpine grassland ecosystems. Based on the zoning results of alpine grasslands, this study proposes a framework of “high-quality protection—moderate-quality enhancement—low-quality restoration,” which holds significant value in the protection and restoration of alpine grasslands (**Figure 5**). The research findings indicate significant differences in spatial distribution, degradation risks, and more among various quality grades of alpine grasslands (**Figure 1**), providing theoretical support and guidance for subsequent protection and restoration strategies. Therefore, this study proposes pathways for the protection and restoration of alpine grasslands from the perspective of different quality grades.

Regarding the protection of HQ grasslands, the findings highlight the crucial need to establish ecological fragile zones and nature reserves (Ma and Yang, 2023), with a focus on preserving pristine, uninhabited grasslands. Given the inherent vulnerability and irreparability of grassland ecosystems (Niu et al., 2022), protective measures should adhere to principles of respecting nature and prioritizing conservation, while assessments of the comprehensive value of economic, environmental, and resource aspects, as well as potential ecological negative impacts, are essential during development and utilization phases. Furthermore, science-based grassland protection plans should be formulated based on locational conditions and resource endowments. In particular, for HQSZ, it is crucial to maintain the current state. For HQPZ, vigilance against degradation risks from disruptive factors is necessary, along with the implementation of improved grazing policies to ensure sustainable development.

In terms of MQ grassland enhancement, the research indicates that implementing a dynamic, multifaceted monitoring framework is necessary, employing various methods and scales to advance integrated monitoring and assessment of alpine grassland ecosystems (Akiyama and Kawamura, 2007). Measures for enhancing moderate-quality grasslands should align with natural principles, leveraging Nature-Based Solutions to harness natural recovery benefits and reduce restoration costs. In QRPZ, natural restoration should be prioritized through measures such as natural diffusion, moderate enclosure, soil and water conservation, and maintaining biodiversity. Moreover, the significance of artificial restoration should also be acknowledged. In QILZ, supplementary interventions such as ecological substrate improvement, artificial planting, and localized irrigation are necessary. This study emphasizes the importance of plant species selection and pastoral community involvement in the success of natural recovery processes, asserting that success should not only be measured through vegetation recovery but also by assessing whether the original attributes and structures of the ecosystem have been restored, thereby fostering natural recovery and achieving a positive feedback loop (Nilsson et al., 2016).

In the context of LQ grassland restoration, referencing HQ grasslands is crucial. The restoration of LQ grasslands should involve cultivating suitable forage species, promoting forage demonstration projects, and developing long-term management plans. When selecting grass species, priorities should be given to those with strong adaptability, drought resistance, and cold tolerance, such as sand *alfafa* and wild barley, to improve vegetation recovery capacity (Porqueddu et al., 2016). Forage demonstration projects can enhance pastoral community engagement and awareness by establishing model sites, implementing windbreaks, and providing various technical training. This study provides specific implementation plans for the restoration of low-quality grasslands, highlighting the synergistic effects of multiple factors as essential for successful restoration. Continuous monitoring, evaluation, and policy support, alongside risk management strategies and ecological service payment mechanisms, are vital for ensuring the long-term effectiveness and sustainable development of grassland restoration. Furthermore, fostering interdepartmental cooperation can ensure information sharing and strategic coordination, optimizing grassland resource management and promoting comprehensive recovery and steady development of grassland ecosystems.

The zoning framework proposed in this study offers a structured approach to managing changes in grassland quality by considering the resilience and pressures of different regions, while adapting to varying environmental conditions. However, the limitations of this framework must also be acknowledged. It relies on current quality assessments, which may not fully capture the temporal ecological dynamics induced by ongoing climate change and anthropogenic pressures. Furthermore, although the framework is adaptive, it has not fully integrated ecological initiatives such as natural grassland protection, grazing reduction, the establishment of Qiangtang National Park, and relocations from extremely high-altitude areas. Thus, future efforts require continuous policy adjustments and localized implementation strategies. Adaptive zoning is closely aligned with Nature-Based Solutions, leveraging natural processes and ecosystem restoration to achieve ecological balance. Through strategic zoning policies that prioritize natural recovery alongside moderate human interventions, this study addresses the degradation of alpine grasslands and contributes to sustainable grassland management practices.

## 5 Conclusion

This study provides a systematic assessment and analysis of the quality, resilience, and pressures of alpine grassland ecosystems, emphasizing the critical role of adaptive zoning management in the protection and restoration of alpine grassland quality. By constructing the alpine grassland quality and evaluating its resilience and pressures, we revealed the spatial heterogeneity of alpine grasslands and proposed specific practical adaptive zoning management strategies tailored for different ecological regions. The results indicate that priority should be given to the protection of high-quality grasslands to prevent ecological degradation. For moderate-quality grasslands, we recommend the implementation of dynamic enhancement measures that promote natural recovery and improve ecological functions. The restoration of low-quality grasslands requires a targeted approach focusing on the introduction of suitable plant species and the establishment of long-term management mechanisms supported by local communities. By clarifying the characteristics and needs of different grassland qualities, this study provides new insights into adaptive management, offering scientific evidence and implementation pathways for the sustainable management of alpine grasslands in the Tibetan Plateau. This aims to effectively address increasingly complex ecological challenges and ensure regional ecological security.

## Data Availability

The data analyzed in this study is subject to the following licenses/restrictions: The data that support the findings of this study are available from the corresponding author upon reasonable request. Requests to access these datasets should be directed to Shurong Zhang srzhang@bnu.edu.cn.
