# Supplementary material for: Adaptive management for alpine grassland of the Tibetan Plateau based on a multi-criteria assessment
Source: Front Plant Sci. 2025 Mar 12;16:1518721. doi: 10.3389/fpls.2025.1518721 (PMC11937058; doi:10.3389/fpls.2025.1518721)
Supplement: Supplementary file 1 [file DataSheet1.docx]

**Supporting information**

| Table S1 Weights of Grassland Quality Evaluation Indicators | | | | |
| --- | --- | --- | --- | --- |
|  | Alpine meadow | | Alpine steppe | |
|  | Comprehensive score | Weight | Comprehensive score | Weight |
| Above-ground biomass | 0.194 | 0.120 | 0.069 | 0.043 |
| Below-ground biomass | 0.058 | 0.036 | 0.118 | 0.074 |
| Vegetation cover | 0.209 | 0.129 | 0.175 | 0.109 |
| Simpson index | 0.210 | 0.130 | 0.173 | 0.108 |
| Shannon-Wiener index | 0.213 | 0.132 | 0.171 | 0.107 |
| Pielou index | 0.172 | 0.106 | 0.162 | 0.101 |
| Soil pH | 0.103 | 0.064 | 0.072 | 0.045 |
| Soil Organic carbon | 0.122 | 0.075 | 0.147 | 0.092 |
| Total nitrogen | 0.131 | 0.081 | 0.112 | 0.070 |
| Total phosphorus | 0.011 | 0.007 | 0.162 | 0.101 |
| Available nitrogen | 0.093 | 0.057 | 0.173 | 0.108 |
| Available phosphorus | 0.001 | 0.001 | 0.007 | 0.004 |
| Soil moisture | 0.063 | 0.039 | 0.006 | 0.004 |
| Soil bulk density | 0.039 | 0.024 | 0.053 | 0.033 |

Table S2 Vegetation index list

|  | Vegetation index | Equation | Source |
| --- | --- | --- | --- |
| 1 | Green Chlorophyll Index (CIg) | CIg=NIR/G-1 | Gitelson et al. (2005) |
| 2 | Red Edge Chlorophyll Index (CIre) | Clre=NIR/RE-1 | Gitelson et al. (2003) |
| 3 | DATT Index (DATT) | DATT=(NIR-RE)/(NIR-R) | Datt (1999) |
| 4 | Difference Vegetation Index (DVI) | DVI=NIR-R | Tucker (1979) |
| 5 | Green Difference Vegetation Index (GDVI) | GDVI=NIR-G | Tucker (1979) |
| 6 | Green No-normalized Different Vegetation Index (GNDVI) | GNDVI=(NIR-G)/(NIR+G) | Gitelson et al. (1996) |
| 7 | Green Re-normalized Different Vegetation Index (GRDVI) | GRDVI=(NIR-G)/sqrt(NIR+G) | Cao et al. (2013) |
| 8 | Green Ratio Vegetation Index (GRVI) | GRVI=NIR/G | Buschmann and Nagel (1993) |
| 9 | Green Soil Adjusted Vegetation Index (GSAVI) | GSAVI=1.5*(NIR-G)/(NIR+G+0.5) | Sripada et al. (2016) |
| 10 | Green Wide Dynamic Range Vegetation Index (GWDRVI) | GWDRVI=(0.12*NIR-G)/(0.12*NIR+G) | Cao et al. (2013) |
| 11 | Modified Chlorophyll Absorption In Reflectance Index1 (MCARI1) | MCARI1=((NIR-RE)-0.2*(NIR-R))*(NIR/RE) | Haboudane et al. (2004) |
| 12 | Modified Chlorophyll Absorption In Reflectance Index 2 (MCARI2) | MCARI2=1.5*(2.5*(NIR-R)-1.3*(NIR-RE))/sqrt((2*NIR+1)2-(6*NIR-5*sqrt(R)-0.5) | Haboudane et al. (2004) |
| 13 | Modified Double Difference Index (MDD) | MDD=(NIR-RE)/(RE-G) | Lu et al. (2017) |
| 14 | Modified Enhanced Vegetation Index (MEVI) | MEVI=2.5* (NIR-RE)/(NIR+6*RE-7.5*G+1) | Cao et al. (2013) |
| 15 | Modified Normalized Difference Index (MNDI) | MNDI=(NIR-RE)/(NIR-G) | Cao et al. (2013) |
| 16 | Modified Normalized Difference Red Edge (MNDRE) | MNDRE=(NIR-RE-2*G)/(NIR+RE-2*G) | Cao et al. (2013) |
| 17 | Modified Red Edge Transformed Vegetation Index (MRETVI) | MRETVI=1.2*(1.2*(NIR-R)-2.5*(RE-R)) | Lu et al. (2017) |
| 18 | Modified Simple Ratio (MSR) | MSR=(NIR/R-1)/sqrt(NIR/R+1) | Chen (1996) |
| 19 | Modified Green Simple Ratio (MSR_G) | MSR_G=(NIR/G-1)/sqrt(NIR/G+1) | Cao et al. (2013) |
| 20 | Modified Red Edge Simple Ratio (MSR_RE) | MSR_RE=(NIR/RE-1)/sqrt(NIR/RE+1) | Lu et al. (2017) |
| 21 | MERIS Terrestrial Chlorophyll Index (MTCI) | MTCI=(NIR-RE)/(RE-R) | Dash and Curran (2004) |
| 22 | Normalized Difference Red Edge (NDRE) | NDRE=(NIR-RE)/(NIR+RE) | Barnes et al. (2000) |
| 23 | Normalized Difference Vegetation Index (NDVI) | NDVI=(NIR-R)/(NIR+R) | Rouse et al. (1974) |
| 24 | Normalized Green Index (NGI) | NGI=G/(NIR+G+RE) | Sripada et al. (2006) |
| 25 | Normalized NIR Index (NNIR) | NNIR=NIR/(NIR+R+RE) | Sripada et al. (2006) |
| 26 | Normalized Red Edge Index (NREI) | NREI=RE/(NIR+G+RE) | Cao et al. (2013) |
| 27 | Greeen optimized Soil Adjusted Vegetation Index (GOSAVI) | GOSAVI=(NIR–R)/(NIR+R+0.16) | Cao et al. (2013) |
| 28 | SR | SR=RE/R | Shahi et al. (2023) |
| 29 | Normalized Red Index (NRI) | NRI=R/(NIR+R+RE) | Lu et al. (2017) |
| 30 | Renormalized Difference Vegetation Index (RDVI) | RDVI=(NIR-R)/sqrt(NIR+R) | Roujean and Breon (1995) |
| 31 | Red Edge Difference Vegetation Index (REDVI) | REDVI=(NIR-RE) | Cao et al. (2013) |
| 32 | Red Edge Normalized Difference Vegetation Index (RENDVI) | RENDVI=(NIR-RE)/(NIR+RE) | Elsayed et al. (2015) |
| 33 | Red Edge Ratio Vegetation Index (RERVI) | RERVI=(NIR/RE) | Gitelson et al. (1996) |
| 34 | Red Edge Optimal Soil Adjusted Vegetation Index (REOSAVI) | REOSAVI=1.5*(NIR-RE)/(NIR+RE+0.5) | Cao et al. (2013) |
| 35 | Red Edge Transformed Vegetation Index (RETVI) | RETVI=0.5*(120*(NIR-R)-200*(RE-R)) | Lu et al. (2017) |
| 36 | Red Edge Wide Dynamic Range Vegetation Index (REWDRVI) | REWDRVI=(0.12*NIR-R)/(0.12*NIR+R) | Cao et al. (2013) |
| 37 | Ratio Vegetation Index (RVI) | RVI=NIR/R | Jordan et al. (1969) |
| 38 | Soil-Adjusted Vegetation Index (SAVI) | SAVI=1.5*(NIR-R)/(NIR+R+0.5) | Huete et al. (1988) |
| 39 | Transformed Normalized Vegetation Index (TNDVI) | TNDVI=sqrt((NIR-R)/(NIR+R)+0.5) | Sandham and Zietsman (1997) |
| 40 | Optimal Vegetation Index (VIopt) | VIopt=1.45*(NIR^2+1)/(R+0.45) | Reyniers et al. (2006) |
| 41 | Wide Dynamic Range Vegetation Index (WDRVI) | WDRVI=(0.12*NIR-R)/(0.12*NIR+R) | Gitelson (2004) |
| 42 | Optimized Soil Adjusted Vegetation Index (OSAVI) | OSAVI=(1 + 0.16) * (NIR - R) / (NIR + R + 0.16) | Cao et al. (2013) |
| 43 | B | B | / |
| 44 | G | G | / |
| 45 | R | R | / |
| 46 | RE | RE | / |
| 47 | NIR | NIR | / |

Table S3 Multiple stepwise regression

|  |  | Nonnormalized coefficient | | Standardization coefficient | t | P | VIF | R² | Adjust R² | F |
| --- | --- | --- | --- | --- | --- | --- | --- | --- | --- | --- |
|  |  | B | SE | Beta |  |  |  |  |  |  |
| AM | Constant | 0.395 | 0.117 | 0 | 3.361 | 0.001** | - | 0.46 | 0.446 | F=40.115 P=0.000** |
|  | RENDVI | 0.36 | 0.116 | 0.39 | 3.1 | 0.003** | 2.777 |  |  |  |
|  | MNDRE | -0.21 | 0.082 | -0.323 | -2.563 | 0.012* | 2.777 |  |  |  |
| AS | Constant | -1.358 | 0.245 | 0 | -5.54 | 0.000** | - | 0.63 | 0.612 | F=32.026 P=0.000** |
|  | VIopt | 0.29 | 0.07 | 0.478 | 4.126 | 0.000** | 2.042 |  |  |  |
|  | REOSAVI | 0.631 | 0.141 | 0.778 | 4.486 | 0.000** | 4.58 |  |  |  |
|  | nir | 0.881 | 0.277 | 0.507 | 3.186 | 0.002** | 3.85 |  |  |  |

Note: ** and * represent 1% and 5% significance levels respectively

AM: GQI=0.395+0.36*RENDVI-0.21*MNDRE R^2^=0.446

AS: GQI=-1.358+0.29*VIopt+0.631* REOSAVI+0.881*nir R^2^=0.612

Table S4 Grassland quality index inversion model of UAV multispectral index

| Grassland type | Model class | Model Equation | R^2^ |
| --- | --- | --- | --- |
| AM | linearity | GQI=0.6473*RENDVI+0.0821 | 0.43578894 |
|  | twice | GQI =-0.7132*RENDVI^2+1.3919*RENDVI-0.0904 | 0.45953359 |
|  | logarithm | GQI =0.289*ln(RENDVI)+0.6324 | 0.45327943 |
|  | power | GQI =0.7832*RENDVI^1.0552 | 0.60496755 |
|  | exponent | GQI =0.1188*e^(RENDVI*2.1563) | 0.48428823 |
| AS | linearity | GQI =0.4277*VIopt-0.7224 | 0.49905684 |
|  | twice | GQI =0.4127*VIopt^2-1.6957*VIopt+1.9871 | 0.51828661 |
|  | logarithm | GQI =1.0851*ln(VIopt)-0.6434 | 0.49079573 |
|  | power | GQI =0.0198*VIopt^3.0486 | 0.36059874 |
|  | exponent | GQI =0.0161*e^(VIopt*1.1958) | 0.36307221 |

The specific expression is as follows:

AM: GQI =0.7832*RENDVI^1.0552 R^2^=0.446

AS: GQI =0.4127*VIopt^2-1.6957*VIopt+1.9871 R^2^=0.612

Table S5 Partial least squares regression model

| Grassland type |  | Constant | LAI | NDVI | EVI |
| --- | --- | --- | --- | --- | --- |
| AM | GQI | 0.467 | 0.028 | 0.088 | -0.026 |
| AS | GQI | 0.355 | 0.047 | -0.116 | 0.138 |


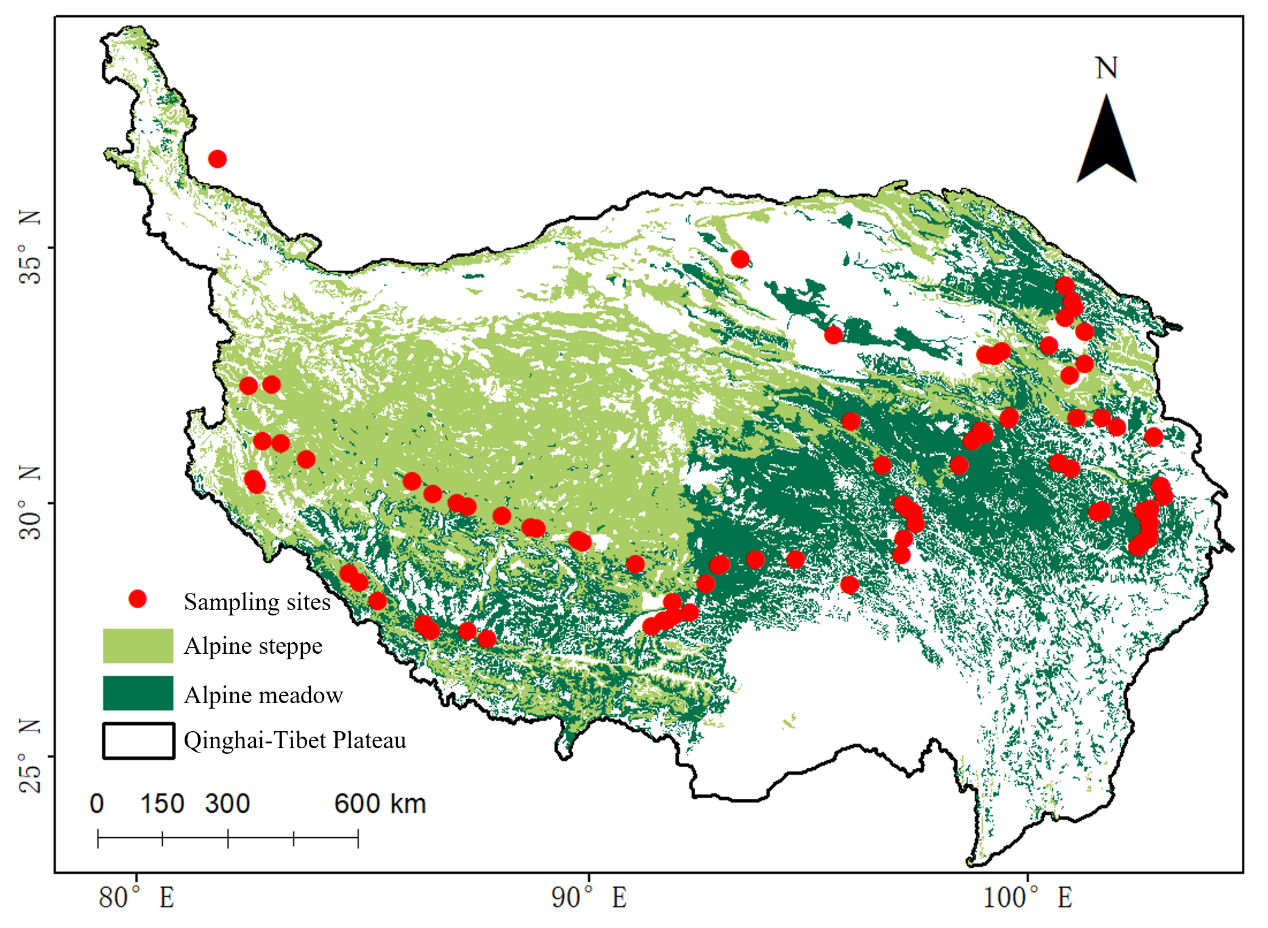


Fig. S1. Location of the sampling sites.


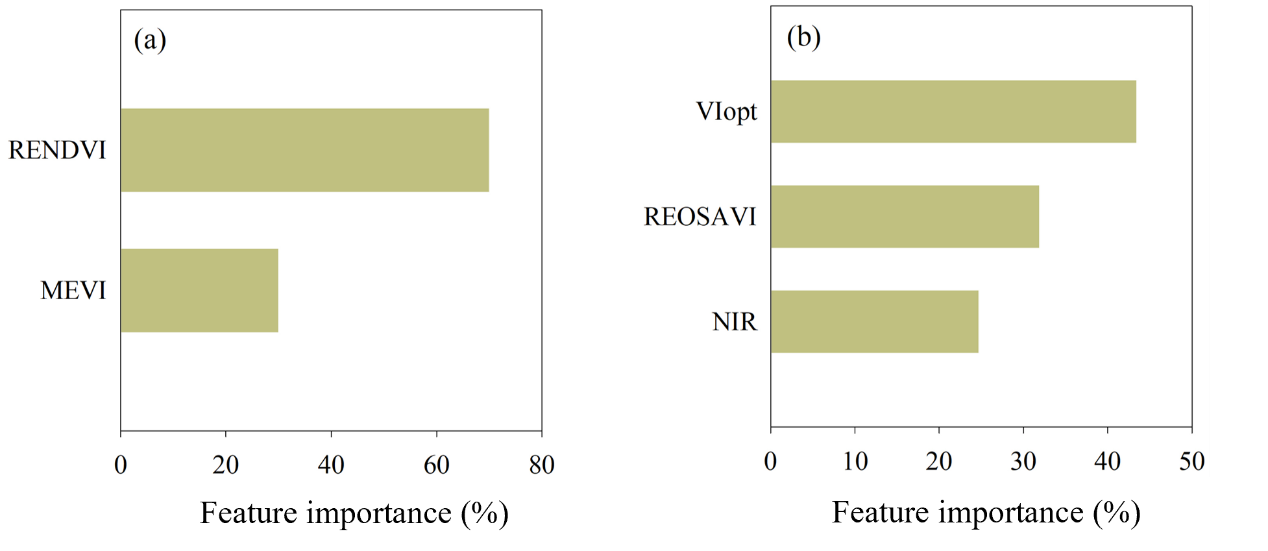


Fig. S2. Importance of vegetation index: (a) alpine meadow;(b) alpine steppe.


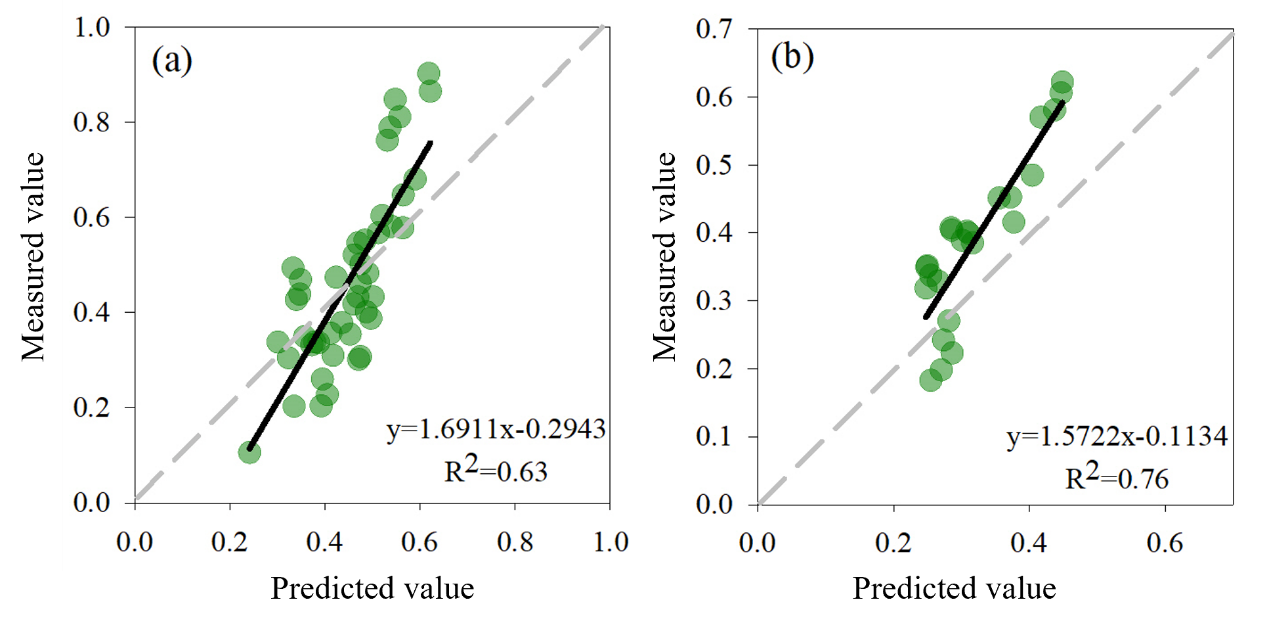


Fig. S3. Validation of landscape scale grassland vegetation quality index model: (a)alpine meadow; (b): alpine steppe


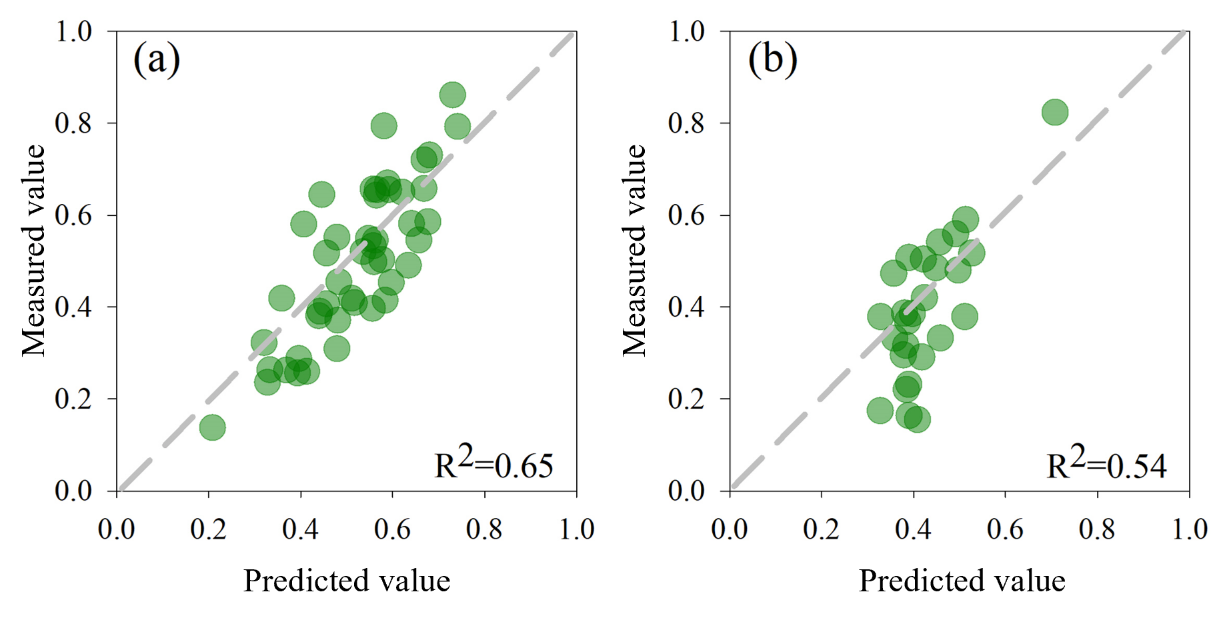


Fig. S4. Validation of regional scale grassland quality index model: (a) alpine meadow; (b) alpine steppe.
